# 3D Printed Hydrogels for Ocular Wound Healing

**DOI:** 10.3390/biomedicines10071562

**Published:** 2022-06-30

**Authors:** Mohamadreza Aghamirsalim, Mohammadmahdi Mobaraki, Madjid Soltani, Mohammad Kiani Shahvandi, Mahmoud Jabbarvand, Elham Afzali, Kaamran Raahemifar

**Affiliations:** 1 Translational Ophthalmology Research Center, Tehran University of Medical Science, Tehran 14176-14411, Iran; aghamirsalim@gmail.com (M.A.); jabarvand@tums.ac.ir (M.J.); 2 Department of Biomedical Engineering, Amirkabir University of Technology, Tehran P.O. Box 15875-4413, Iran; m.mahdimobaraki70@gmail.com; 3 Department of Mechanical Engineering, K. N. Toosi University of Technology, Tehran 19967-15433, Iran; mohammadkiani941@gmail.com; 4 Department of Electrical and Computer Engineering, University of Waterloo, Waterloo, ON N2L 3G1, Canada; 5 Advanced Bioengineering Initiative Center, Multidisciplinary International Complex, K. N. Toosi University of Technology, Tehran 14176-14411, Iran; 6 Centre for Biotechnology and Bioengineering (CBB), University of Waterloo, Waterloo, ON N2L 3G1, Canada; 7 Pharmaceutics Research Center, Institute of Neuropharmacology, Kerman University of Medical Science, Kerman 76169-13555, Iran; af_el35@yahoo.com; 8 Data Science and Artificial Intelligence Program, College of Information Sciences and Technology (IST), Penn State University, State College, PA 16801, USA; kraahemi@gmail.com; 9 Department of Chemical Engineering, University of Waterloo, 200 University Avenue West, Waterloo, ON N2L 3G1, Canada; 10 Faculty of Science, School of Optometry and Vision Science, University of Waterloo, 200 University Avenue West, Waterloo, ON N2L 3G1, Canada

**Keywords:** 3D bioprinting, ocular wound healing, scaffold-based hydrogels, bioink

## Abstract

Corneal disease is one of the most significant causes of blindness around the world. Presently, corneal transplantation is the only way to treat cornea blindness. It should be noted that the amount of cornea that people donate is so much less than that required (1:70). Therefore, scientists have tried to resolve this problem with tissue engineering and regenerative medicine. Fabricating cornea with traditional methods is difficult due to their unique properties, such as transparency and geometry. Bioprinting is a technology based on additive manufacturing that can use different biomaterials as bioink for tissue engineering, and the emergence of 3D bioprinting presents a clear possibility to overcome this problem. This new technology requires special materials for printing scaffolds with acceptable biocompatibility. Hydrogels have received significant attention in the past 50 years, and they have been distinguished from other materials because of their unique and outstanding properties. Therefore, hydrogels could be a good bioink for the bioprinting of different scaffolds for corneal tissue engineering. In this review, we discuss the use of different types of hydrogel for bioink for corneal tissue engineering and various methods that have been used for bioprinting. Furthermore, the properties of hydrogels and different types of hydrogels are described.

## 1. Introduction

Human eyes have a complex anatomy, and the main structures include the following: tear film, cornea, anterior chamber, iris, pupil, crystalline lens, vitreous, retina, and optic nerve. The cornea is a transparent window in the front part of the human eye that covers the anterior chamber, iris, and pupil and is primarily responsible for focusing light rays onto the retina. As a result, the cornea plays a crucial role in the optic system because it accounts for approximately two-thirds of the human eye [1]. The healing processes of the cornea can be divided into five distinct components: cell death, migration, proliferation, differentiation, and extracellular matrix remodeling. Nearly 10 million patients around the world are suffering from corneal blindness. Therefore, there is an increasing clinical need for scaffolds as cornea grafts for utilization in corneal transplantation. Regenerative medicine and tissue engineering have shown acceptable properties that can affect patients’ treatment [2,3]. Consequently, three-dimensional (3D) scaffolds with properties similar to cornea tissue have the potential to replace donor tissue for transplantation or to improve wound healing [4].

3D printing is a useful technology for fabricating artificial scaffolds and has been developed to print 3D structures layer-by-layer. It should be mentioned that, in order to achieve outstanding structure and bioengineered tissues, various areas of science such as material science, computational science, and biology should be integrated [5]. With this technology, various types of complex structure can be created. Additionally, cells or growth factors can be printed during the process to enhance the biocompatibility of the structure. Biomaterials that can be used for the bioprinting of various types of scaffolds are known as bioink. Bioink must have diverse properties, as follows: biocompatibility, printable, adjustable degradation properties, decent swelling properties, etc. Hydrogels have received considerable attention in the past 50 years, and they have been distinguished from other materials because of their unique and outstanding properties. Researchers have defined hydrogels in several different ways, but most scientists are in agreement that these types of biomaterials have three-dimensional hydrophilic polymer networks that can entrap considerable amounts of water or biological fluids but will not dissolve in water [6,7]. Furthermore, hydrogels can be used to create a highly hydrated 3D environment for different types of cells. Generally, natural hydrogels such as chitosan, alginate collagen, etc. are utilized for tissue engineering, but these types of hydrogels have some weaknesses, such as their mechanical properties. An ideal scaffold for corneal tissue engineering should be transparent and have good mechanical properties and outstanding biocompatibility because, with these properties, the scaffold allows cell proliferation, adherence, and migration [8,9]. All in all, natural hydrogels are a good candidate for cornea tissue engineering.

Here, we provide a review of recent progress toward 3D-printed scaffold-based hydrogels for corneal tissue engineering. Furthermore, different methods of bioprinting scaffolds for the cornea are discussed. Moreover, the properties of hydrogels, different types of hydrogel, and their pros and cons are explained.

## 2. Anatomy of the Cornea

**Figure 1:**
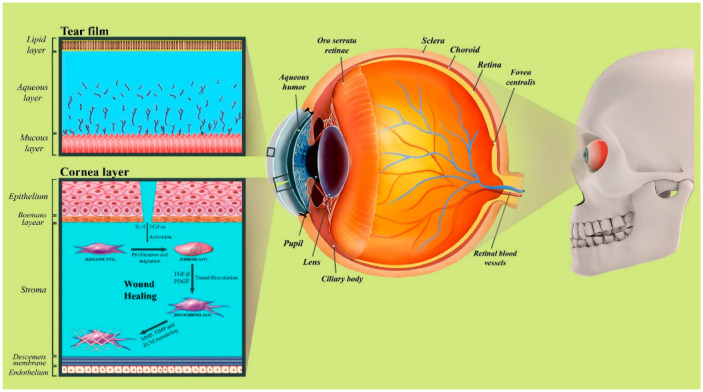
The anatomical structure of the eye and cornea tissue in the human cornea wound healing mechanism. Photoshop 2020 was used to create this figure.

The human eye has a complex anatomy. The main structures of the eye include the following: tear film, cornea, anterior chamber, iris, pupil, crystalline lens, vitreous, retina, and optic nerve. The tear film is a transparent thin fluid layer approximately 3 μm thick and 3 μL in volume, covering the ocular surface, which consists of lacrimal fluid and the secretion of the meibomian and conjunctival glands. Tear film is made up of three layers: First, the most superficial layer of the tear film is the oily layer (or lipid layer), which is derived principally from the meibomian glands in the lid margin. It is a thin layer (approximately 50 to 100 nm) that contains many different lipid species, including polar lipids that interact with the aqueous layer and non-polar lipids that make up its bulk [10]. The principal role of this layer is the avoidance of evaporation of the lower layers and the reduction of surface friction between the lid and the ocular surface [11]. The aqueous layer is the second layer, which is a watery lacrimal fluid that forms the bulk of the tears and contains a specific variety of proteins, such as bactericidal lysozyme, electrolytes, and water. Most of the aqueous fluid is secreted by the lacrimal gland and the accessory glands of Krause and Wolfring. The last layer, the deepest and densest layer of the tear film, is called the mucin layer (or mucous layer) and is derived from the conjunctival goblet cells, as well as some secretion from the lacrimal gland [12] (Figure 1).

The cornea is a transparent window in the front part of the human eye that covers the anterior chamber, iris, and pupil and is primarily responsible for focusing light rays onto the retina. The cornea accounts for approximately two-thirds of the human eye’s total focusing power (approximately 43 diopters); in addition, it serves as a mechanical and chemical barrier protecting the inner eye. The Epithelium, Bowman’s layer, stroma, Descemet’s membrane, and endothelium are the five layers of the human cornea [1]. The epithelium layer is the most superficial layer of the cornea and is composed of approximately 5–7 layers of non-keratinized stratified squamous epithelium cells, which are shed constantly on the exposed layer and are regenerated by multiplication in the basal layer. Irregularity of the corneal epithelium disrupts the smoothness of the air/tear-film interface, which results in reducing visual acuity [13]. The Bowman’s Layer is a tough second layer that can be described as a condensed, acellular region of the apical stroma, comprised primarily of randomly organized yet tightly woven collagen fibrils. The stroma lies underneath the Bowman’s Layer and accounts for approximately 90% of the corneal thickness [1]. The stroma layer is a transparent layer consisting of approximately 200 layers of mainly type I and type V collagen fibrils and extracellular matrix, produced, organized, and maintained by corneal stromal keratocytes. The lattice arrangements of the collagen fibrils in the stroma reduce light scattering. The Descemet’s membrane is composed mainly of collagen type IV fibrils and serves as the modified basement membrane of the corneal endothelium [14]. This layer is a thin acellular layer around 5–20 μm thick, depending on the age of the person. The monolayered corneal endothelium is responsible for regulating solute and fluid transport between the corneal stromal compartments and the aqueous layer and for maintaining corneal transparency. If the endothelium can no longer keep a proper fluid balance, stromal overhydration due to excess fluids and subsequent loss of stroma transparency will occur [15].

The processes involved in the healing of corneal disruption can be divided into five distinct components: cell death, migration, proliferation, differentiation, and extracellular matrix remodeling [16]. Many similarities can be observed in the healing processes of different parts of the cornea. Limbal stem cells and remodeling of the basement membrane are principal parts of corneal epithelial healing. The sequence of transformation of stromal keratocytes to fibroblasts and myofibroblasts is a major process in stromal wound healing. Many growth factors and cytokines have an important influence on regulating the stimulation of growth, proliferation, migration, differentiation, adhesion, ECM deposition, and proteinase regulation of the cells involved in wound healing [17].

## 3. Bioprinting Techniques Classification

**Figure 2:**
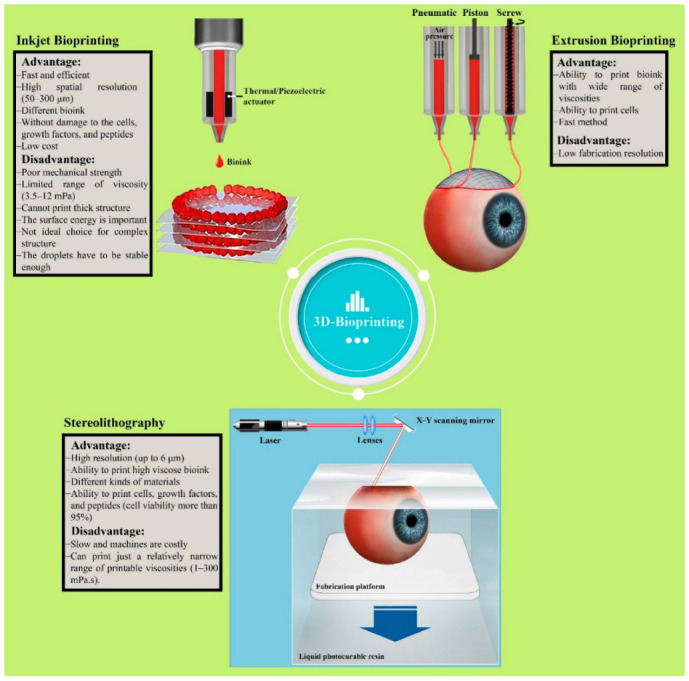
Schematic of different bioprinting methods with their advantage and disadvantage. Photoshop 2020 was used to create this figure.

There are various methods, such as stereolithography [5], inkjet [18], microextrusion [19], fuse deposition modeling [20], etc., used for printing different structures, and all of them have certain pros and cons. Although all these methods are useful and beneficial, only stereolithography, inkjet, and microextrusion have been used for printing biological materials to biofabricate scaffolds for tissues or organs. Different parameters, such as cell viability, mechanical properties, resolution, cost, etc., have a direct effect on choosing the best methods [21]. In the following section, we will discuss these three methods (Figure 2).

### 3.1. Stereolithography

Stereolithography (SLA), or laser-based bioprinting, was used in the 1980s for the first time, and it is one of the earliest methods for printing various types of materials [22]. This established 3D printing technique is one of the most attractive methods for printing specific 3D geometries such as multilayer scaffolds and biological material, including live cells, DNA, and peptides [23,24]. As a result, this method has been extensively developed for tissue engineering during the last decade, because many researchers have reported the cell viability of this method as being almost 100% [25,26,27]. The SLA technique consist of three main components: a layer for absorbing laser energy, the donor slide, and the pulsated laser beam source. In SLA, a high energy light source (UV light) or long-wavelength laser are utilized so as to cure and harden liquid polymers at exact locations [28]. Although this method is slow and not very affordable, the resolution of the scaffolds is outstanding (up to 6 μm), and various types of materials can be printed [29].

### 3.2. Inkjet Bioprinting

The inkjet printing method, also known as drop-by-drop or material jetting 3D printer, is one of the eldest methods for printing biological and non-biological materials. This method has the ability to print low-viscosity biomaterials on the substrate in small fractions of nearly 1–100 picolitres [30]. Thermal heaters [31] and piezoelectric actuators [32] are two different strategies that are used for printing various types of biomaterials because the bioink in inkjet printing is deposited in the form of droplets, continuous or not [33].

Inkjet printing is not only a fast method for printing complex structures, but it is also the most efficient method for printing various types of bioinks. Moreover, this method has the flexibility to be used for printing very difficult structures for tissue engineering [34], and with this useful strategy, different live cells, growth factors, and peptides can be printed alone or together in the same construct. It should be noted that the resolution of the scaffold is reasonable, but the mechanical properties of these types of scaffolds are weak, which is a negative point of this method [29].

### 3.3. Extrusion Bioprinting

Extrusion bioprinting, or direct writing, is one of the most attractive strategies for printing scaffolds for tissue engineering, and mechanical force or pneumatic are two methods for extruding bioink. It should be noted that the viscosity of the bioink is important in this method, and the ink utilized in extrusion bioprinting should be from 30 to 6 × 10^7^ mPa. Therefore, this strategy of printing has received important attention in the world of tissue engineering because bioink with a wide range of viscosities can be used to print. Additionally, another important property of this method is its ability to print bioink with great cell densities, and this advantage is useful for printing scaffolds for medical applications. It should be noted that, due to existing shear stresses and bioink deformation during the printing process, cell viability is decreased. On the other hand, with this method, different types of hydrogels can be printed easily. Although this method, in comparison to other methods, is fast, the resolution of the structure is not outstanding (~200 μm) [35].

## 4. Hydrogel

Hydrophilic gels, called hydrogels, have received considerable attention in the past 50 years, and they have been distinguished from other materials because of their unique and outstanding properties. Researchers have defined hydrogels in several different ways, but most scientists are in agreement that these types of biomaterials have three-dimensional hydrophilic polymer networks that can entrap considerable amounts of water or biological fluids but will not dissolve in water. The reason for this property is the existing different hydrophilic functional groups, such as –OH, –CONH, –CONH_2_, –SO_3_, and –COOH, that are attached to the polymer backbone in the polymer chain, and the presence of physical and/or chemical crosslinks [36].

**Figure 3:**
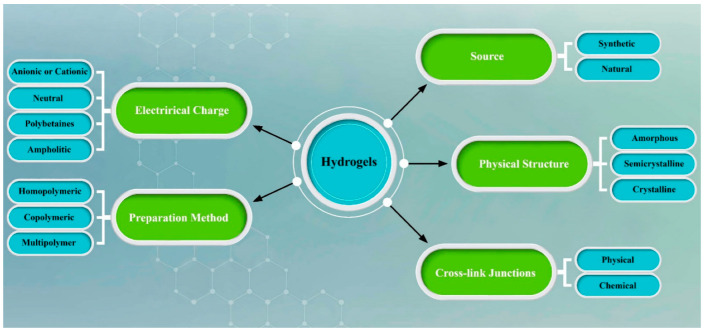
Schematic of hydrogels based on electrical charge, preparation method, source, physical structure, and cross-link junction. Photoshop 2020 was used to create this figure.

There are various methods for the preparation of different hydrogels, such as copolymerization, cross-link, or free-radical polymerization. Generally, all hydrogels consist of three different parts: monomer, initiator, and cross-linker, and as a result hydrogen bonding, covalent bonds, electrostatic interactions, and van der Waals interactions are created due to existing cross-linkers in the structure. All in all, due to the hydrophilic functional groups in hydrogel biomaterials, the amount of water that can be entrapped is not comparable to polymer structures [37,38]. Water in the hydrogel and water could exist in three different states. Water does not have any bonds with polymer functional groups and is called free water. It should be noted that the amount of free water existing in the hydrogel has a direct relationship with the structure of the hydrogel. As an illustration, the amount of free water in a compact structure is not very much in comparison with soft hydrogels. In the next phases, semi-bound water and polymer functional groups create weak interactions, and finally, in the last step, bonding between polymer and water hydrogen is created [39] Moreover, softness, and smartness are the other unique properties of these types of materials. As a result, over the last few decades, due to these properties, these types of materials have been used in different areas, such as biomedical applications [40], tissue engineering and regenerative medicines [30], drug delivery systems [41], pharmaceuticals [42], wound dressing [43], diagnostics [44], and biosensors [45]. Generally, hydrogels can be divided into different categories, such as electrical charge, preparation method, source, physical structure, and cross-link junction (Figure 3).

### 4.1. General Properties of Hydrogels

Hydrogels are a special group of materials that present as neither completely liquid nor completely solid, and they mimic three-dimensional tissue environments [46]. Physical and chemical cross linking methods are two different strategies for the gelation of various types of hydrogels [47]. Although interaction between polymer networks in terms of physical strategy is weak, in chemical strategy, strong bonds are created inside the hydrogel, and consequently the hydrogel resists permanent deformation [48,49]. On the other hand, because cells can have a decent interaction with the matrix of hydrogels, various studies have been carried out over the past 20 years to characterize the properties of hydrogels used for biological research. Most researchers emphasize that properties such as ligand density, stiffness, and network porosity have a direct effect on cell behavior [50].

Elasticity and swelling properties are two important factors of hydrogels, and it should be mentioned that the cross-link and charge densities of the polymer network are two various parameters that have a direct effect on them. Moreover, the polymer network concentration has an important effect on the elasticity and swelling properties. It is important to understand that the stiffness and network porosity of the hydrogels has a significant effect on the cell fate, and before using hydrogels for biological entities (proteins and cells), this parameter should be considered. On the other hand, enhancing ionic groups in the structure of hydrogels causes an increase in the swelling capacity of the hydrogel. The reason for this phenomenon is enhanced counterions and, as a result, increasing osmotic pressure inside the gel [46].

Degradation is another important property of hydrogels used for tissue engineering because, during degradation, tissue has to be replaced. Two different methods (hydrolytically and/or enzymatically) are suggested for degradation of the hydrogels with time. Consequently, various research has been carried out during the last decade concerning controlling degradation time. Degradation of hydrogels leads to changes in most of the properties of hydrogels, such as their mechanical properties [43].

### 4.2. Classification of Hydrogel

**Figure 4:**
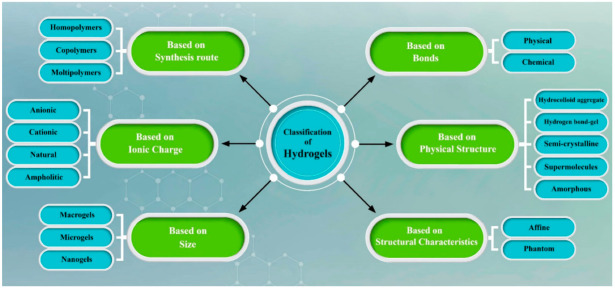
Schematic of classification of hydrogels. Photoshop 2020 was used to create this figure.

Although various categories have been suggested for the classification of hydrogels, usually hydrogels can be categorized as detailed below (Figure 4):

#### 4.2.1. Based on Physical Structure and Chemical Composition

Classification of the hydrogels based on configuration can be classified as follows:

(A) Non-crystalline or amorphous hydrogels;
(B) Crystalline hydrogels;
(C) Semicrystalline hydrogel—a group of hydrogels that consist of one amorphous phase and one crystalline phase [51].

#### 4.2.2. Based on Polymer Composition

There are three different types of hydrogel that have been utilized as biomaterials:

(A) Homopolymeric hydrogels—a single class of monomer is used to prepare this kind of hydrogel [52];
(B) Copolymeric hydrogels—when two or more different monomers are utilized for the preparation of the hydrogel, this type of hydrogel is called a copolymeric hydrogel. It should be mentioned that at least one of the monomers has to be a hydrophilic component [53];
(C) Multipolymer Interpenetrating Polymeric (IPN) hydrogel—IPN hydrogels are another group of hydrogel that utilize two synthesized and/or natural polymers, where one is cross-linked and the other is non-cross-linked [54].

#### 4.2.3. Based on Type of Cross-Linking

Hydrogels could be classified into two categories:

(A) Chemical cross-linked—hydrogels with chemical cross-links that have permanent interactions;
(B) Physical cross-linked—hydrogels with physical networks that have temporary junctions, such as hydrogen bonds, ionic interactions, or hydrophobic interactions [55].

#### 4.2.4. Classification Based on Source

Two different sources exist for preparing hydrogels: natural and synthetic polymers.

(A) Natural:
  Recently, natural polymer sources have been replaced by synthetic materials because these types of materials have outstanding properties such as decent biocompatibility, brilliant cellular and tissue responses, acceptable biodegradability, and no toxic production throughout the degradation process. While this group of materials has decent properties, there are some disadvantages, such as poor mechanical strength, high degradation rate, etc. The most popular natural hydrogels used for ocular wound healing are chitosan, gelatin, collagen, alginate, silk fibroin, hyaluronic acid, etc. [56];
(B) Synthesize
  Synthetic polymers have been attracting attention because of their exceptional properties, such as satisfactory mechanical properties, controllable degradation rate, and tunable geometry. These types of materials have no natural source, and they are the result of a polymeric reaction. The most attractive synthesized polymers used for synthesizing hydrogels as biomaterials include Hydroxyethyl methacrylate (HEMA), N-isopropyl AAm (NIPAAm), Poly (ethylene glycol) (PEG), Methacrylic acid, Methoxyethyl methacrylate (MEMA), etc. [57].

## 5. Natural Polymers as Bioink for Corneal Tissue Engineering

The bioink and the 3D printing technology used are two important parameters for the bioprinting of biomaterials and organs. Hydrogels as a bioink are the best applicant because these types of materials can not only prepare a decent environment for cell proliferation, but their structure is also similar to the natural extracellular matrix. Additionally, because natural hydrogels such as chitosan [58,59], collagen [60], and gelatin [61] show decent biocompatibility, biodegradability, swelling properties, cytocompatibility, etc., they have received extensive attention during the last few decades. Although natural hydrogels have outstanding properties for bioprinting and tissue engineering, these types of hydrogels have limitations, such as weak mechanical properties. On the other hand, synthesized hydrogels also have good biocompatibility, but degradation is an important issue that should be considered before using them [62].

### 5.1. Gelatin

Gelatin is a biodegradable polypeptide derived from the partial hydrolysis of collagen. Collagen maintains the integrity of connective tissues, such as cartilage, corneas, tendons, ligaments, blood vessels, and dentin. However, collagen has low antigenicity, which arises from the determinant polypeptide structures in the three spiral chains as well as the central areas of the molecules. This drawback has greatly limited its application in biomedical fields [63].

Gelatin is a natural water-soluble polymer that can absorb 5–10 times the equivalent weight of water. By increasing the temperature, the dissolution speed can be accelerated to a certain degree. Gelatin solution is amphoteric due to alkaline amino acids and acid functional groups. Different isoelectric points of gelatin solution stem from the processing protocols used for gelatin extraction [64]. Depending on the temperature, pH, concentration, and preparation method, gelatin solutions manifest different properties. Gelatin solutions can be gelled at low temperature (approximately 20–30 °C) by cooling to form hydrogels, which is a sol-gel transition process. In the gelation process, gelatin molecules begin to be arranged locally with hydrogen, electrostatic, and hydrophobic bonds [65]. As a result of these non-specific bonds, the hydrogel becomes thermo-sensitive. Gelatin-based hydrogels can be printed and accumulated by a computer-aided design (CAD) model [66].

Gelatin methacrylate (GelMA) is a cost-effective, easy to synthesis, biocompatible potential bioink for biofabrication applications. Moreover, the mixture of GelMA with the photo-initiator can impose rapid crosslinking during and after extrusion while exposed to UV light [67]. GelMA also has appropriate biological properties due to the cell adhesive RGD (Arginine, glycine, and aspartate) patterns and MMP-degradable amino acid chain. Therefore, it can stimulate the adhesion, spreading, and proliferation of different cell types [63]. This polymer is also useful for corneal tissue engineering. Consequently, these types of materials have been used, alone or combined with others, for cornea tissue. As an example, in 2020, Zhonga et al. [68], for subconjunctival ocular injection, used GelMA hydrogel as a bioink to print structures with tunable mechanical properties. The purpose of using this type of hydrogel was the encapsulation of conjunctival stem cells, and it showed outstanding viability and the ability to preserve stem cell behavior. In this research, scientists used DLP-based rapid bioprinting for printing hydrogels with injectable properties. In another study in this year, Chen et al. used a pneumatic extrusion system to fabricate GelMA hydrogel scaffolds that were reinforced with poly (ε-caprolactone)-poly (ethylene glycol) to achieve a 3D fiber hydrogel construct [69]. To have scaffolds with similar properties to a native corneal stroma structure, the fiber spacing was adjusted (six dissimilar fiber spacings were tested), and they found that the direct writing of dissimilar fiber topological (ranging from 50 to 500 μm) structures had an effect on various properties, such as mass swelling ratio, light transmittance, and mechanical strength. Consequently, it was seen that fiber spacing is an important factor that has direct effects on all properties of the whole construct, and they found that the design of the fiber in the hydrogel structure can encourage the regeneration of damaged corneal stroma, both in vitro and in vivo. Moreover, Bektas et al. used GelMA hydrogels to design a 3D bio-printed corneal stroma [70]. For this purpose, by the extrusion method, cell-loaded 3D-printed hydrogels with stromal keratocyte cells were printed. As a result, the hydrogels enhanced cell viability (98%), and only an 8% lose weight was seen after three weeks in the degradation test. Bio-printed hydrogels not only had acceptable mechanical properties after three weeks, but the transparency of the cell-loaded and cell-free hydrogels was also outstanding (over 80%).

In another recent study, Mahdavi et al. used the stereolithography printing method for printing the similar dome-shaped structure of the human corneal stroma [71]. In this study, two different concentrations of GelMa (7.5% and 12.5%) were used as bioink, and as expected, due to high concentration, the scaffolds with 12.5% GelMa had better mechanical properties in comparison to those with 7.5%. Additionally, increasing the concentration of the scaffolds not only enhanced optical transmittance, but water content was also similar to native corneal stroma tissue. The important result of this research was the effect of concentration on the cytocompatibility test. By increasing the concentration of the GelMa, cytocompatibility enhanced meaningfully. GelMa is not only a useful bioink for printing scaffolds for corneal tissue engineering, but it is also beneficial for cell-loaded scaffolds. As an illustration, this type of hydrogel was used as a bioink for printing corneal stroma equivalent to substitute for the native tissue, and different conditions of printing, such as the spindle speed and the nozzle speed in the x-y direction, were changed so as to find the best sample for the native tissue [70]. The most important properties of these scaffolds were their outstanding degradation rate, and only 8% weight loss was seen after three weeks. In this research, keratocyte was chosen as the cell; cell viability was 98% on day 21, and as a result, these cells with a 3D-printed hydrogels structure had outstanding biocompatibility. Moreover, the mechanical properties of the structure were similar to cornea tissue, and transparency was over 80% in comparison to the native cornea. In another study in 2020, poly (2-hydroxyethyl methacrylate) (pHEMA) was added so as to enhance the mechanical properties of interpenetrating network hydrogels [9]. pHEMA is a useful polymer that is usually utilized for soft contact lenses; when incorporating it into the hydrogel, not only are the mechanical properties of the hydrogels enhanced meaningfully, but the cell viability and transparency of the hydrogels are also increased. It should be mentioned that, recently, a group of researchers improved the properties of GelMa hydrogel by sequential hybrid (physical followed by UV) crosslinking called GelMA+ [72]. This improved material in comparison with regular GelMA has better mechanical properties, and these properties were thermally stable at physiological temperatures. As a result, these types of hydrogels were used as nano-patterned substrates by Rizwan et al. for corneal tissue engineering application. They found GelMA+ not only had outstanding biodegradation kinetics, but it could also be used as a cell-carrier device. Therefore, due to the important and unique properties of this type of GelMa hydrogel, GelMa+, can be used as a useful bioink for the 3D printing of thin film for corneal tissue engineering.

### 5.2. Collagen

**Figure 5:**
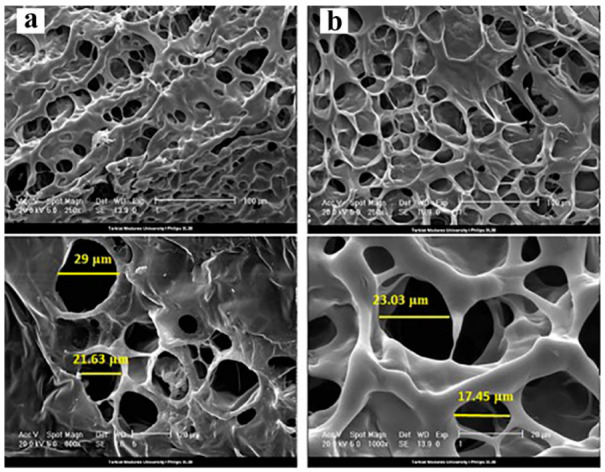
SEM of the surface morphology of (**a**) collagen-gelatin hydrogels with average diameters between 10 and 30 μm and (**b**) gelatin hydrogels with average diameters between 20 and 30 μm [74]. It should be mentioned that section (**a**,**b**) are used with permission from [74]. Copyright Elsevier, 2019.

Collagen, a natural protein in the human body, consists of structural proteins in the ECM of several tissues. Collagens have several structural and hierarchical organizations that are distributed in different animal tissues. Type I collagen is the most present type in bone tissue, skin, tendons, ligaments, and the cornea [73], while type II and IV are typically present in cartilage and basement membranes. Collagens composed of polypeptide chains comprised of different amino acids are followed by tripeptides glycine X-Y (X and Y indicate proline and hydroxyproline, respectively). Collagen sequenced by Y is known as the most exclusive collagen and is used for the identification and quantification of collagens in extracts [65]. Various research has been carried out regarding collagen for corneal tissue engineering. As an illustration, in 2019, Goodarzi et al. used Type-I collagen–gelatin hydrogel for corneal tissue engineering [74]. In this study, N-(3-Dimethylaminopropyl)-N′-ethylcarbodiamide hydrochloride (EDC) and N-hydroxysuccinimide (NHS) were utilized as cross-linkers, and human bone-marrow mesenchymal stem cells (hBM-MSCs) were used as cell evaluations. In this study, the effect of the percentage of collagen on the different properties of gelatin hydrogel was investigated. For example, it was found that composite hydrogel had better transparency than gelatin-based hydrogels and collagen-gelatin had an outstanding porous structure and, as a result, better cell attachment was achieved (Figure 5).

Collagen-based materials are suitable for bioprinting; however, they have limitations, such as liquid remaining at low temperatures and the formation of a fibrous structure with increased temperature or neutral pH [75]. Several studies have been carried out to develop methods to enhance the printability of collagen-based bioinks. Combining hybrid collagen and synthetic polymers, and printing collagen into a sacrificial support gel are some of the methods used to improve the bioink’s rheological properties [76].

Every bioink has its own printing parameters to achieve 3D geometries with the desired accuracy and resolution. Bioink viscosity and stiffness should ensure structure reproducibility. Collagen, though, has low viscosity, so most of the developed approaches require low temperatures to increase its viscosity and ability to print solid frameworks. However, bioactive molecules or cells can be added to improve such properties. Pure collagen can jellify at pH 7.4 and 37 °C, but it is unstable over time [77]. The crosslinking procedures for COL require the use of toxic chemical agents that can limit cell viability. Optimization of the crosslinking concentrations or using natural agents with reduced toxicity, such as genipin, will increase cell viability [73]. To enhance the ink’s rheological specification for printing, viscosity-enhancing components have been mixed with GelMA-precursor solution, for example, gellan gum or hyaluronic acid, as well as co-deposition with reinforcing biomaterials [63].

Creating personally-specific implantable 3D bio-constructs with highly controlled dimensions and geometries needs the bioink to be stable within its functional timeframe. Therefore, to avoid shape loss and undesirable properties such as swelling, shrinkage, and degradation, optimization of the properties is required [78]. For this purpose, Isaacson et al. utilized sodium alginate and type I collagen as a bioink using extrusion methods to print artificial cornea [79]. Scientist at Newcastle University utilized suitable support structures to print native human corneal stroma. The low-viscosity bioink had the ability to encapsulate corneal keratocytes cells for 7 days. Additionally, different percentages of collagen were used to find the best viscosity and acceptable stiff properties for corneal tissue. Moreover, the concentration of the collagen in this research was changed in order to improve the printability of the bioink. On the other hand, alginate improved the transparency properties. In another study, collagen-based hydrogels were used as a bioink for creating corneal stromal [80]. The optical properties of this bio-printed structure were not only similar to native corneal stromal, but the mechanical properties of the hydrogel were also ideal for cornea tissue engineering. Additionally, corneal stromal keratocytes were used and printed with hydrogels as 3D biomimetic models. It should be mentioned that, due to the outstanding properties of this bioink, corneal stromal keratocytes maintained their native keratocyte phenotypes after 7 days. Kutlehria et al. used calcium chloride for crosslinking gelatin–collagen bioink as a bio-printed structure for corneal stromal equivalents [81]. In this study, stereolithography and extrusion methods were used to print the scaffolds. As in other studies, human corneal keratocyte cells were incorporated in the bioink to print a cell-laden structure. After two weeks, cells could maintain great viability (>95%). Additionally, the scaffolds showed outstanding transparency and smoothness, and they possessed the required curvature. Moreover, Wu et al., by extrusion techniques, successfully printed cell-laden constructs based on collagen–gelatin [82]. By adding collagen, the degradation properties of the alginate were improved, and for mimicking corneal tissue, a structure with a macro-porous network has printed. Because of these properties, the viability of human corneal epithelial cells was outstanding, and the cells that were printed in the structure could grow faster.

### 5.3. Others Polymers

Silk fibroin is an essential protein originating from the silkworm. For example, because of the ability to mimic natural tissue frameworks, silk-based structures could regulate cell phenotypes. Additionally, by printing composite scaffolds based on the silk and other natural/synthesized polymers, the biocompatibility and bioactivity of 3D printing scaffolds are significantly improved. On the other hand, the rough morphology and stiffness of the scaffolds can be adjusted by changing the percentage of the silk. Additionally, by adjusting the amount of drugs, cells, and growth factors that encapsulate during the 3D printed process, the viscosity, biocompatibility, and rheology properties of silk bioink is controllable [83]. Due to these unique and exceptional properties of silk, these material-based scaffolds are a great potential candidate for bioink in tissue engineering and regenerative medicine [77]. 

In one study, Gong et al. utilized a two-step method for formulating silk fibroin hydrogel as a new kind of bioink with outstanding mechanical properties and biocompatibility for tissue engineering [84]. A double-network hydrogel with acceptable shear-thinning behaviors, great strength, and good resilience for 3D printing was synthesized. In this study, for example, so as to test the mechanical properties of the printed hydrogel, 2 kg weights were placed on the 12 mm-diameter scaffolds, and they were not destroyed. Therefore, this kind of hydrogel based on natural polymers is an attractive candidate for tissue engineering. In another study, Farasatkia et al. synthesized a transparent silk/GelMA film for cornea tissue engineering and regeneration [85]. In this study, to enhance the transparency, mechanical properties, degradation rate, and swelling ratio of the film, different volume ratios of two polymers were tested, and the best ratio was found to be 30% silk and 70% GelMA. In this specific combination, the mechanical properties of the hybrid films were acceptable; the main reason for this phenomenon were the existing silk nanofibrils in the composite. Additionally, the film could absorb water up to 138 ± 27%, and it should be mentioned that a content of more than 30% silk had a direct effect on the degradation rate of hybrid films.

For corneal stromal tissue engineering, Kim et al., by the extrusion bioprinting method, fabricated a transplantable transparent cornea structure [86]. The bioink of this study was decellularized corneal extracellular matrix hydrogel, and by changing the flow rate and nozzle diameter, the shear stress could be changed. Additionally, the viscosity of the bioink has a direct effect on the shear stress, and the bioink in this study had shear-thinning properties. It should be noted that, during the extrusion process, shear stress has an important effect on cell fate. Therefore, the cell behaviors of corneal keratocyte have been observed under four dissimilar conditions and after 28 days; each group showed diverse cellular morphology. As a result, this aligned collagenous structure created a lattice pattern similar to the structure of a native human cornea after 4 weeks in vivo. In another study in 2019, Kim et al. again used a cornea-derived decellularized extracellular matrix as a bioink because of its outstanding transparency, great biocompatibility, ability to encapsulate cells, and lack of cytotoxicity [87]. Nearly all cells (human turbinate mesenchymal stem cells) that encapsulated in the bioink in this study remained alive. Using in vivo models for one-month and two-month periods, the hydrogels were found to be biocompatible with mice and rabbit cornea. Researchers found that this type of bioink has properties and safety elements similar to clinical-grade collagen. Finally, can also be recommended for different types of corneal disease. 

## 6. Smart Hydrogels

**Figure 6:**
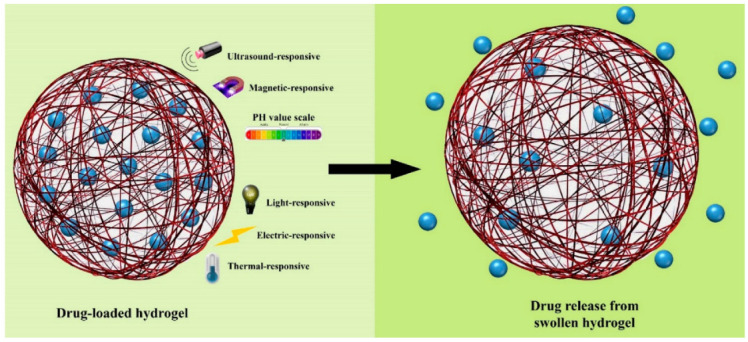
Parameters that have an effect on the properties of smart hydrogels. Photoshop 2020 was used to create this figure.

Over the past few decades, different research has been carried out in the field of hydrogels in order to characterize and understand their properties for use as biomaterials. Recently, significant attention has been drawn to smart hydrogels, and these types of hydrogel are very important because smart hydrogels can respond when located in different environments. As an illustration, smart hydrogels show dramatic changes in properties, such as sol–gel transition, swelling behavior, mechanical properties, network structure, etc., when parameters such as temperature, pH, ionic strength, electromagnetic radiation, and some others are changed [38]. It should be mentioned that pH- and temperature-sensitive hydrogels (thermoresponsive) are two important groups of hydrogel that are widely used for biological application and as biomaterials, because not only are these two elements controllable under in vivo and in vitro conditions, but pH and temperature are also actors that have physiological significance inside the human body (Figure 6) [88]. Generally, smart hydrogels have been used for controlled drug delivery into various parts of the body and organs, biomedical applications, biosensor, regenerative medicine, and tissue engineering [89].

The most useful smart hydrogels used for cornea tissue engineering are thermo-responsive hydrogels. The perfect thermo-responsive hydrogel for tissue engineering is liquid at room temperature, and when it is located at 37 °C (temperature of the body) turns into gel form. Because of this property, this type of hydrogel forms the most important group of smart hydrogels for biomedical applications. As a result, thermo-responsive hydrogel is useful for the delivery of various types of drugs and for its ability to control cell adhesion or detachment; it can also be used in injectable gels, etc. Although thermo-responsive hydrogels are useful for biomedical applications, their mechanical properties and toxicity are two important issues that scientists have tried to resolve [90,91].

**Figure 7:**
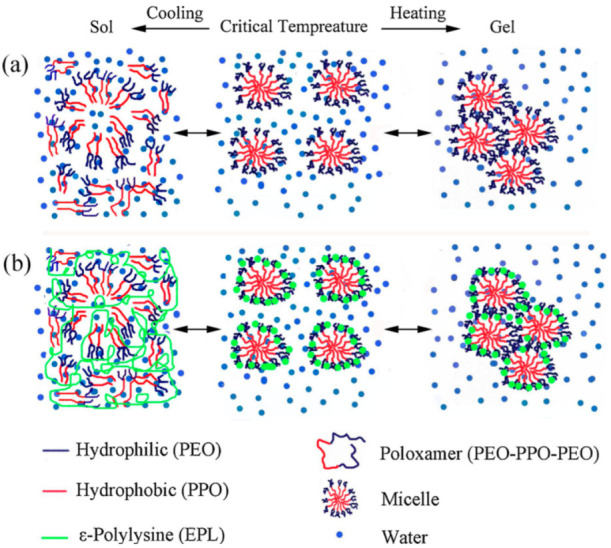
Mechanism of gelation without ε-Polylysine (EPL) (**a**), and with ε-Polylysine (EPL) (**b**) [91]. It should be mentioned that section (**a**,**b**) are used with permission from [92]. Copyright Elsevier, 2020.

Different types of thermo-responsive hydrogels have been developed for corneal tissue engineering because of their unique properties. For example, corneal neovascularization is one of the main reasons for severe disorders on the surface of the ocular. For this purpose, in 2020, Xu et al. used poloxamer-based thermo-responsive hydrogel for sustained release of bone morphogenetic protein 4 (BMP4) [92]. Additionally, ε-Polylysine (EPL) was added to provide temperature sensitive properties. The aim of this research was to inhibit corneal neovascularization after eye operations, and the phase transition temperature of the hydrogel was from 22 °C to 25 °C (Figure 7). Therefore, at normal physiological temperature, the liquid form of the thermo-responsive hydrogel is transformed in order for it to gel very quickly. In another study, the triblock copolymer of poly(D,L-lactic-co-glycolic acid)-block-poly(ethylene glycol)-block-poly(D,L-lactic-co-glycolic acid) (PLGA-PEG-PLGA) was utilized as a thermo-responsive hydrogel in order to dissolve corneal neovascularization [92]. In this study, Liu et al. used metformin (MET) and levofloxacin hydrochloride (LFH) as drugs because all of the polymers and drugs could dissolve in water at room temperature. They found that drugs could release sustainably for one month, and this kind of hydrogel is useful for anti-angiogenic therapy.

## 7. Conclusions

Corneal disease is one of the most significant reasons for blindness all around the world, and corneal transplantation is the only way to treat corneal blindness. It should be mentioned that the amount of cornea that people donate is so much less than that required (1:70). For resolving this important and crucial problem, solutions such as tissue engineering and regenerative medicine have emerged. The main goal of tissue engineering and regenerative medicine is to repair the function of damaged tissues. It should be noted that fabricating scaffolds for the cornea is difficult due to their unique properties, such as transparency and geometry. Traditional methods are not suitable for fabricating all types of scaffold, but 3D bioprinting is a method that can print different complex scaffolds with different geometries, and as a result, its emergence presents a clear possibility for overcoming this problem. Biomaterials that are used for bioprinting various types of scaffolds are known as bioink. Bioink must have different properties, such as biocompatibility, printability, adjustable degradation, decent swelling, etc. Hydrogels have received considerable attention in the past 50 years, and they have been distinguished from other materials because of their unique and outstanding properties. Consequently, hydrogels are a worthy bioink for the bioprinting of different scaffolds for corneal tissue engineering. With hydrogels as a bioink, not only can scaffolds with different geometries be printed, but cells and growth factors can also be printed.

## Data Availability

Data sharing not applicable.
